# Procalcitonin as diagnostic marker of infection in solid tumors patients with fever

**DOI:** 10.1038/srep28090

**Published:** 2016-06-17

**Authors:** B. Vincenzi, I. Fioroni, F. Pantano, S. Angeletti, G. Dicuonzo, A. Zoccoli, D. Santini, G. Tonini

**Affiliations:** 1 Medical Oncology, University Campus Bio-Medico of Rome, Rome, Italy; 2 Clinical Pathology and Microbiology Laboratory, University Campus Bio-Medico of Rome, Rome, Italy

## Abstract

In oncologic patients fever is a non-specific clinical marker of different clinical settings. Procalcitonin (PCT) seems to be the most promising infection marker. We aimed to define the potential role of PCT as an earlier diagnostic marker in patients with fever and solid tumor. This retrospective study enrolled 431 patients. All of them performed hemoculture (HE) and basal PCT assessment (reference laboratory cut-off: ≤0.5 or >0.5 ng/dL) before starting antibiotic therapy. Gram positive (G+), negative (G−) or Fungi infection were detected. A statistically significant difference in PCT levels between patients with positive and negative HE was observed (P < 0.0001). Moreover comparing PCT values in patients with positive and negative HE, we obtain in the positive HE subpopulation an AUC of 0.7 and a cut-off of 1.52 ng/dL reached high sensitivity (61.6%) and specificity (70.1%). Using this last cut-off, instead of the normal reference value, we achieve a risk reduction to overestimate an infection status of 23.4%. We support the clinic usefulness of serum PCT dosage in febrile advanced solid tumor patients. A PCT cut-off of 1.52 ng/dL could be helpful in the management of the antibiotic therapy preventing delays of oncologic treatments.

Oncologic patients are at high risk for developing infections; this could lead to hospitalization, interruptions in therapy schedules, and even death. Neutropenia is recognized as one of the most serious hematologic toxicity during cancer treatment with chemotherapy and radiotherapy and it is a very common risk factor of infections in oncologic patients. Other important predisposing factors to bacterial infections are skin and mucosal barrier disruption, dysfunction of cell-mediated immunity, obstruction of bronchial tree, biliary tract, urinary tract and gastrointestinal tract, central nervous system dysfunction and some diagnostic and therapeutic invasive procedures^1^. In oncologic patients, fever is a non-specific clinical marker and it could represent a sign of several different clinical settings such as drugs reaction, real infection status or a paraneoplastic syndrome called also “neoplastic fever”^2^. It is necessary to recognize an infection or a non-infection status earlier^3^. Nowadays, among several known bio-markers, procalcitonin (ProCT) seems the most promising^4^. ProCT is a hormokine composed of 116 amino acids and it is the precursor of the hormone calcitonin composed of only 33 amino acids^5^. A generalized release of ProCT as expression of inflammatory reaction can be induced through the direct stress of bacterial toxins or indirectly through the humoral host or cell-mediated inflammatory response^6^. ProCT value increases rapidly within 2–4 hours from the onset of a bacterial infection^7^. It has an half-life of 22–24 hours and therefore its concentration may be halved daily when infection is resolving^8^. ProCT rises mostly during Gram-negative systemic infections and its concentration reflects bacterial load^9^. ProCT in oncologic neutropenic febrile patients was examinated in several studies but a recent meta-analysis failed to define its specific role^10^. Only few studies regarding non-neutropenic oncologic patients were performed^11^. In this population the onset of an unknown origin fever could lead a diagnostic issue. Therefore Shomali *et al.* explored ProCT role as a bio-marker to differentiate an infectious origin fever and a non-infectious origin fever in non-neutropenic patients with both solid tumors and hematological malignancies^12^. Our study aimed to defining the potential role of ProCT, used as an earlier diagnostics biomarker^13^,^14^, in a cohort of patients with fever and with diagnosed solid tumor followed in our oncologic ceter consecutively.

## Patients and Methods

### Patients’ population

This is a single institution, retrospective, observational study. The investigations were performed after approval by the Ethic Committee of the University Hospital Campus Bio-Medico of Rome and all experiments were performed in accordance with relevant guidelines and regulations. 431 consecutive patients were enrolled; all of them presented a known diagnosis of solid metastatic or locally advanced tumor (not operable) and were admitted to Medical Oncology Department of Campus Bio-Medico University Hospital of Rome between January 2009 and March 2013 with fever (>38.3 °C or 2 consecutive >38 °C readings). All patients performed hemoculture and basal PCT assessment before starting any empirical antibiotic therapy. Previous antibiotic treatment started within 4 weeks before hospital admission was considered an exclusion criterion. Patients was stratified for positive or negative hemoculture (bacteremia or fungemia) and PCT according to the normal reference laboratory cut-off (PCT value ≤0,5 ng/dL or >0.5 ng/dL). Patients with positive hemoculture were further categorized according to the presence of a Gram positive (G+), Gram negative (G−) or Fungi infection.

### Test methods

Each blood culture comprised three sets (time 0, time 30 and time 60) of one aerobic and one anaerobic broth bottles (Bactec Plus Aerobic/F, Becton Dickinson) per patient drawn during 1 h period from cases of clinically suspected bloodstream infection. Blood culture vials were incubated in the Bactec 9240 automated system (Becton Dickinson). From positive broths, subcultures were prepared and, according to the appearance of colonies on subculture plates, the isolates were identified and the antimicrobial susceptibility test performed by Vitek 2.0 compact instrument (bioMerieux sa, Marcy l’ Etoile, France) or Phoenix (Becton Dickinson) instrument with the support of some additional phenotypic tests (such as coagulase test, PYR test and oxidase test). The significance of possible contaminants of blood cultures was assessed by different personnel and according to standard criteria. Laboratory confirmed bloodstream infection had to meet at least one of the following criteria: the patient had a recognized pathogen cultured from one or more blood cultures or the patient has systemic signs of infection (defined as meeting the SIRS criteria) and a common skin contaminant (e.g., coagulase-negative Staphylococci or micrococci) cultured from two or more blood cultures drawn on separate occasions. PCT was assessed in our central laboratory by an immunoluminometric assay. PCT plasma concentrations were measured by an automated Kryptor analyzer, using a time-resolved amplified cryptate emission (TRACE) technology assay (Kryptor PCT; Brahms AG; Hennigsdorf, Germany), with commercially available immunoluminometric assays (Brahms)^15^. PCT was measured at the same moment that a blood culture was delivered to the laboratory. The analytical characteristics of the assays are summarized in Table 1.

### Statistical Methods

A ROC analysis was performed in order to identify the optimal cut-off point for PCT values in G+, G− and Fungi hemoculture positive patients. Area under the curve (AUC) was also computed. Non parametric Mann-Whitney test was used in order to assess statistically significance difference in PCT value between the patients’ subgroups. P values <0.05 were considered to be significant. Statistical analysis was performed using SPSS software (vers. 17).

## Results

### Population’ characteristics

This retrospective, observational study enrolled 431 patients. The population included 235 male and 196 female patients and every patients were at least 18 years old. 343 patients were affected by the most common tumors such as: colon-rectal cancer (80 patients), pancreatic cancer (53 patients), non small cell lung cancer (NSCLC, 49 patients), breast cancer (42 patients), ovarian cancer (23 patients), biliary tract cancer (21 patients), gastric cancer (19 patients), small cell lung cancer (SCLC, 16 patients), bladder cancer (15 patients), kidney cancer (15 patients) and prostate cancer (10 patients). All others 88 patients had rarer cancers. The majority of patients showed distant metastasis (368 patients) and only 63 patients presented locally advanced not operable tumors. Among the whole population, 89 patients had clinical or radiological evidence of localized bacterial infection such as pneumonia, cholangitis, abscess and urinary tract infection (Table 2).

### Cut-off analysis: standard local laboratory value

**Figure 1:**
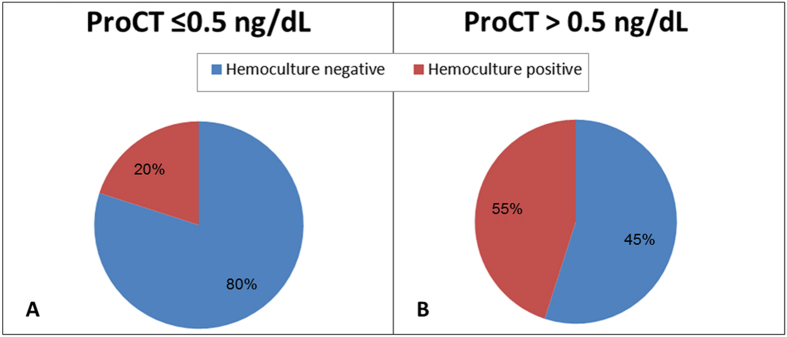
(**A**) Hemoculture stratification for PCT ≤0.5 ng/dL patients; (**B**) Hemoculture stratification for PCT >0.5 ng/dL patients.

**Figure 2:**
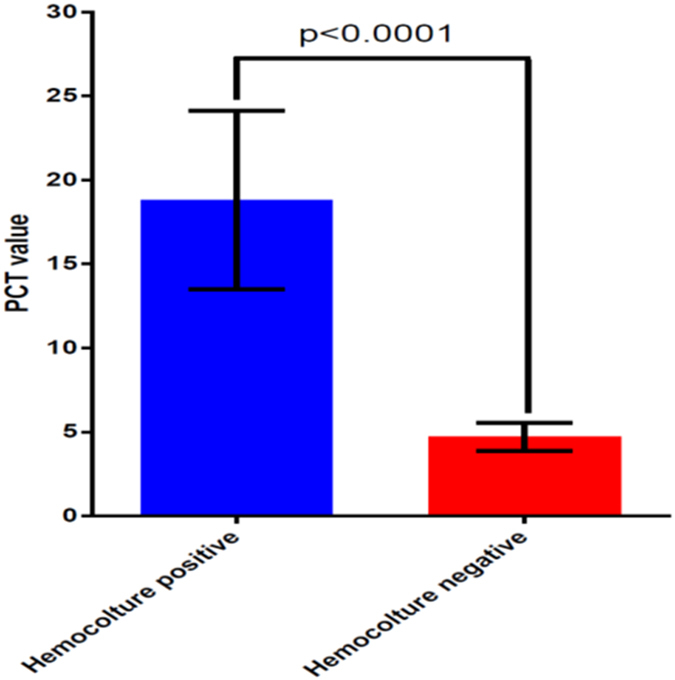
Non parametric Mann-Whitney test results describing difference between hemoculture positive vs. hemoculture negative population.

Among the 431 enrolled solid tumor febrile patients, 181 (42%) showed a positive hemoculture while 250 (58%) a negative one. We perform PCT analysis for each patient and consider a PCT cut-off value of 0.5 ng/dL. Successively we stratified our population into two groups: patients with PCT value ≤0.5 ng/dL and patients with PCT value >0.5 ng/dL. As a result we obtain that 271 patients (62.9%) showed a PCT value >0.5 ng/dL and 160 patients (37.1%) ≤0.5 ng/dL. Analyzing these data more in details we observe that 128 (80%) patients with PCT value ≤0.5 ng/dL presented a negative hemoculture while only 32 (20%) of them showed a positive hemoculture; among patients with PCT value >0.5 ng/dL, 149 (55%) had a positive hemoculture and 122 (45%) a negative one. The PCT median value in the hemoculture positive population was 16.81 (95% CI: 8.294–29.320) vs. 4.72 (95% CI: 3.069–6.365) in the group of negative hemoculture one. Applying the Mann Whitney test we obtain a statistically significant difference in PCT value between the two patients’ subgroups (P < 0.0001) (Figs 1A,B and 2).

Comparing different cancer hystologies no significant differences were detected in terms of PCT median levels; CRC patients were used as control group for median comparisons.

### Cut-off analysis: assessment of new value

**Figure 3:**
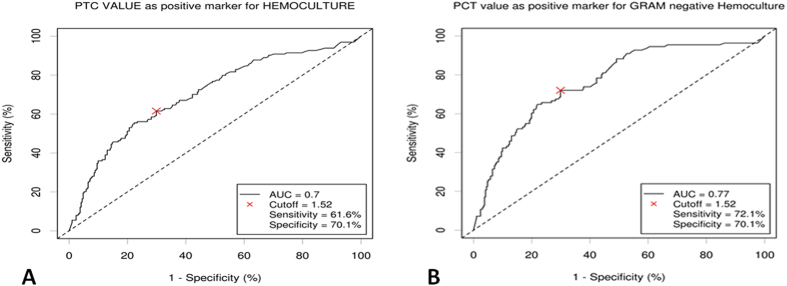
(**A**) ROC curve for various cut-off levels of PCT in differentiating patients with positive and negative hemoculture; (**B**) ROC curve analysis of sensitivity and specificity for the Gram-negative bacteria patients’ group.

Moreover comparing PCT values in patients with positive and negative hemoculture, through the ROC analysis, we demonstrate an AUC of 0.7 and a cut-off value of 1.52 ng/dL in the positive hemoculture subpopulation reached high sensitivity (61.6%) and specificity (70.1%) values (Fig. 3A). Using our new PCT cut-off of 1.52 ng/dL, the false positive rate was 30.4% (patients with PCT value >1.52 ng/dL, but with negative hemoculture), while using the standard PCT cut-off 0.5 ng/dL, the false positive rate was 48.8%. Thus we obtain a reduction of risk of 18.4% to overestimate an infection status in this pattern of patients.

**Figure 4:**
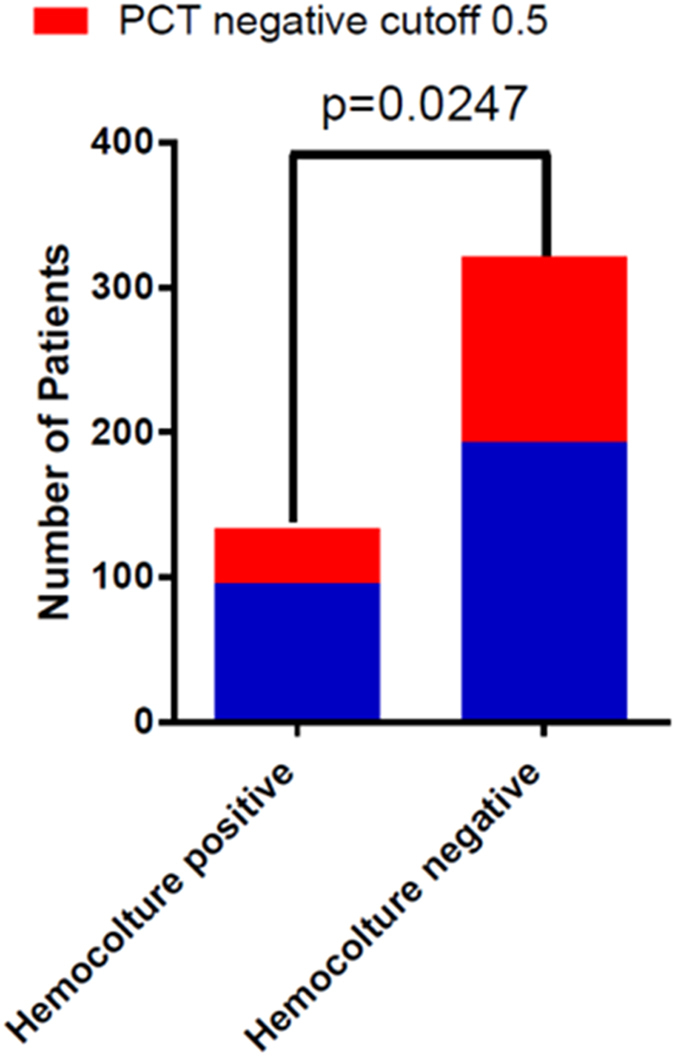
Chi-square statistic for the comparison of AUCs.

The following analysis showed that patients with a positive hemoculture mostly presented a Gram-negative bacteria infection (130 patients (72%)) and only 45 patients (24.8%) had a Gram-positive bacteria infection and only 6 patients (3.3%) showed a funginemia. By these results we tried to identify a more specific and sensitive PCT value cut-off stratifying patients with positive hemoculture into Gram-negative and Gram-positive/fungi isolation: the ROC analysis demonstrates that the PCT cut-off point of 1.52 ng/mL for the Gram-negative bacteria isolations was associated to a sensitivity of 72.1% and specificity of 70.1%, reaching statistical significance (AUC 0.77; P < 0.001) (Fig. 3B). Conversely the PCT cut-off value of the group of patients with a Gram-positive/fungi infection determined by the ROC analysis was 0.575 with a lower sensitivity (49.8%) and lower specificity (50.2%). These results were not statistical significant (AUC 0.5; P < 0.082). Finally, calculation of the Chi-square test was used to value the reliability of both cut-offs: 0.5 ng/dL e 1.52 ng/dL to discriminate true negatives and false negatives patients. We demonstrate that our new PCT cut-off of 1.52 ng/dL seems to be more reliable to identify the negativity of a hemoculture in this solid tumor oncologic population reaching statistical significance (p = 0.0247) (Fig. 4).

## Discussion

The close link between inflammation and cancers has been noticed many years ago. Several chronic inflammatory diseases play a role to promote the onset of a neoplasia such as the inflammatory bowel disease and colon-rectal cancer, asbestosis and mesothelioma, hemochromatosis and hepatocellular carcinoma^16^, Sjogren’s syndrome and lymphoma^17^. On the other hand presence of tumor cells stimulates immune system inducing a permanent inflammatory status which supports cancer cells proliferation, dissemination, inhibition of apoptosis and neo-angiogenesis^18^. We focused our study on the peculiar inflammatory status of oncologic patients. In particular we hypothesize that the basal hyper-procalcitoninemia status in the solid tumor patients, especially during advanced stages of oncologic disease, could probably due to the progressive production of pro-inflammatory cytokines^19^. Bacteremia, which could represents an active infection condition, induces PCT increase. It could justifies why solid tumor patients could benefit from a highest PCT cut-off than non-oncologic patients and even more higher in case of concomitant bacteremia. Our study confirms results of previous studies showing an higher sensitivity and specificity of PCT value in suggesting a Gram-negative infection rather than a Gram-positive infection^20^. The different behavior of PCT in this context was established trough several in vitro studies where human cells were exposed to both Gram+ (lipopolysaccharide, LPS) and Gram- (muramyl dipeptide)^21^ bacterial-derived products. Thus, the LPS that is the major component of the outer membrane of Gram-negative bacteria, promotes secretion of TNF-alpha which is in turn a powerful procalcitonin inductor. This procalcitonin stimulating mechanism does not occur through exposure of muramyl dipeptide, a cell wall component of Gram-positive bacteria^22^,^23^. The “double” influence of both Gram-negative bacteremia and advanced cancer disease on PCT production, could probably influence the PCT cut-off value increasing it until 1.52 ng/dL. Low specificity and sensitivity of PCT value in patients with Gram-positive bacteremia confirms that procalcitonin levels depends on cytokines produced by host immune system in response to the infecting pathogen. In conclusion we support the clinic usefulness of serum procalcitonin dosage in febrile advanced solid tumor patients.

The retrospective nature of the study represent its major limit, hence further prospective study are mandatory to confirm that a PCT cut-off 1.52 ng/dL could be helpful to identify a Gram-negative bacteremia in this specific population. Results from future prospective studies about this cut-off value might be used to help clinicians to decide a prompt start, a continuation or a discontinuance of antibiotic treatment. Finally this findings could prevent unnecessary extensions of antibiotic therapies and patients hospitalization with a consequently undue delays of specific oncologic treatments.

## Additional Information

**How to cite this article**: Vincenzi, B. *et al.* Procalcitonin as diagnostic marker of infection in solid tumors patients with fever. *Sci. Rep.* **6**, 28090; doi: 10.1038/srep28090 (2016).

## Figures and Tables

**Table 1:** Analytical characteristics of PCT measured by an automated Kryptor analyzer, using a time-resolved amplified cryptate emission (TRACE) technology assay (Kryptor PCT;Brahms AG; Hennigsdorf, Germany).

Analytical characteristics of PCT	
Analytical detection limit	0.02 ng/mL
Functional sensitivity	0.06 ng/mL
Measuring range	0.02–50 ng/mL
Intra-assay CV (%)	≤10%
Adult reference limit	0.064 ng/mL

**Table 2:** Demographic and Clinical patients’ characteristics.

Patients’ Characteristics	Patients’ Number	Patients (%)
Age		
18–60 years	149	34.6
61–70 years	126	29.2
>70 years	156	36.2
Gender		
Male	235	54.5
Female	196	45.5
Underlying Cancer		
Colon-rectal cancer	80	18.5
Other gastrointestinal cancers	93	21.6
Thoracic cancers	65	15
Genitourinary cancers	63	14.6
Breast cancer	42	9.7
Others	88	20.6
Cancer Stage		
Locally Advanced Cancer	63	14.6
Distal Metastasis (IV)	368	85.4
Type of infection:		
Urinary tract infections	31	7.2
Cholangitis	28	6.5
Pneumonia	19	4.4
Abscess	11	2.5

## References

[b1] Wingard J. R. & Bowden R. A. Management of Infection in Oncology Patients. Ch. 18–22, 345–430 (Martin Dunitz Taylor and Francis Group, 2003).

[b2] Zell J. A. & Chang J. C. Neoplastic fever: a neglected paraneoplastic syndrome. Support Care Cancer. 13(11), 870–877 (2005). 15864658 10.1007/s00520-005-0825-4

[b3] Browder A.a., Huff J.w. & Petersdorf R.g. The significance of fever in neoplastic disease. Ann Intern Med. 55, 932–942 (1961). 13873560 10.7326/0003-4819-55-6-932

[b4] Tsalik E. L., Jaggers L. B., Glickman S. W., Langley R. J. & van Velkinburgh J. C. *et al.* Discriminative value of inflammatory biomarkers for suspected sepsis. J Emerg Med. 43(1), 97–106 (2012). 22056545 10.1016/j.jemermed.2011.05.072 PMC3740117

[b5] Jullienne A., Segond N., Calmettes C., Moukhtar M. S. & Milhaud G. Biosynthesis of human calcitonin: evidence for a prohormone. Biochem Biophys Res Commun. 95(3), 932–937 (1980). 7417320 10.1016/0006-291x(80)91562-4

[b6] Christ-Crain M. & Müller B. Biomarkers in respiratory tract infections: diagnostic guides to antibiotic prescription, prognostic markers and mediators. Eur Respir J. 30(3), 556–573 (2007). 17766633 10.1183/09031936.00166106

[b7] Gilbert D. N. Use of Plasma Procalcitonin Levels as an Adjunct to Clinical Microbiology. Journal of Clinical Microbiology 48(7), 2325–2329 (2010). 20421436 10.1128/JCM.00655-10 PMC2897488

[b8] Becker K. L., Nylén E. S., White J. C., Müller B. & Snider R. H. Jr. Procalcitonin and the calcitonin gene family of peptides in inflammation, infection, and sepsis: a journey from calcitonin back to its precursors. J Clin Endocrinol Metab. 89(4), 1512–1525 (2004). 15070906 10.1210/jc.2002-021444

[b9] Reinhart K. & Meisner M. Biomarkers in the critically ill patient: procalcitonin. Crit Care Clin. 27(2), 253–263 (2011). 21440200 10.1016/j.ccc.2011.01.002

[b10] Sakr Y., Sponholz C., Tuche F., Brunkhorst F. & Reinhart K. The role of procalcitonin in febrile neutropenic patients: review of the literature. Infection. 36(5), 396–407 (2008). 18759057 10.1007/s15010-008-7374-y

[b11] Ito S., Sato N., Kojika M., Yaegashi Y. & Suzuki Y. *et al.* Serum procalcitonin levels are elevated in esophageal cancer patients with postoperative infectious complications. Eur Surg Res. 37(1), 22–28 (2005). 15818038 10.1159/000083144

[b12] Shomali W., Hachem R., Chaftari A. M., Jiang Y. & Bahu R. *et al.* Can procalcitonin distinguish infectious fever from tumor-related fever in non-neutropenic cancer patients? Cancer. 118(23), 5823–5829 (2012). 22605389 10.1002/cncr.27602

[b13] Schuetz P., Albrich W. & Mueller B. Procalcitonin for diagnosis of infection and guide to antibiotic decisions: past, present and future. BMC Med. 9, 107 (2011). 21936959 10.1186/1741-7015-9-107 PMC3186747

[b14] Póvoa P., Souza-Dantas V. C., Soares M. & Salluh J. F. C-reactive protein in critically ill cancer patients with sepsis: influence of neutropenia. Crit Care. 15(3), R129 (2011). 21595932 10.1186/cc10242 PMC3218995

[b15] Angeletti S., Battistoni F., Fioravanti M., Bernardini S. & Dicuonzo G. Procalcitonin and mid-regional pro-adrenomedullin test combination in sepsis diagnosis. Clin Chem Lab Med. 51(5), 1059–1067 (2013). 23072859 10.1515/cclm-2012-0595

[b16] Ismail F., Mahmoud A., Abdelhaleem H., Mamdoh A. & Geneidy M. *et al.* Primary Sjögren’s syndrome and B-non-Hodgkin lymphoma: role of CD4+ T lymphocytopenia. Rheumatol Int. 33(4), 1021–1025 (2013). 22886469 10.1007/s00296-012-2464-7

[b17] Hussain S. P. & Harris C. C. Inflammation and cancer: an ancient link with novel potentials. Int J Cancer. 121(11), 2373–2380 (2007). 17893866 10.1002/ijc.23173

[b18] Hanahan D. & Weinberg R. Hallmarks of cancer: the next generation. Cell. 13, 646–674 (2011). 21376230 10.1016/j.cell.2011.02.013

[b19] McDonald N. Cancer cachexia and targeting chronic inflammation: a unified approach to cancer treatment and palliative/supportive care. Supportive oncology. 13(4), 157–162 (2007). 17500503

[b20] Charles P. E., Ladoire S., Aho S., Quenot J. P. & Doise J. M. *et al.* Serum procalcitonin elevation in critically Ill patients at the onset of bacteremia caused by either gram negative or gram positive bacteria. BMC Infectious Diseases. 8, 38 (2008). 18366777 10.1186/1471-2334-8-38 PMC2289831

[b21] Tavares E., Maldonado R., Ojeda M. L. & Miñano F. J. Circulating inflammatory mediators during start of fever in differential diagnosis of gram-negative and gram-positive infections in leukopenic rats. Clin Diagn Lab Immunol. 12, 1085–1093 (2005). 16148175 10.1128/CDLI.12.9.1085-1093.2005 PMC1235789

[b22] Skerrett S. J., Liggitt H. D., Hajjar A. M. & Wilson C. B. Cutting edge: Myeloid differentiation factor 88 is essential for pulmonary host defense against Pseudomonas aeruginosa but not Staphylococcus aureus. J Immunol. 172, 3377–3381 (2004). 15004134 10.4049/jimmunol.172.6.3377

[b23] Karlsson H., Larsson P., Wold A. E. & Rudin A. Pattern of cytokine responses to gram-positive and gram-negative commensal bacteria is profoundly changed when monocytes differentiate into dendritic cells. Infect Immun. 72, 2671–2678 (2004). 15102775 10.1128/IAI.72.5.2671-2678.2004 PMC387913

